# Adaptation and Validation of the Well-Being Related to Food Questionnaire (Well-BFQ©) for the French-Speaking General Adult Population of Québec, Canada

**DOI:** 10.3390/nu15051128

**Published:** 2023-02-23

**Authors:** Andrée-Anne Doyon, Alexandra Bédard, Catherine Trudel-Guy, Louise Corneau, Simone Lemieux

**Affiliations:** 1 Centre Nutrition, Santé et Société (NUTRISS), Institut Sur La Nutrition et Les Aliments Fonctionnels, Université Laval, Québec, QC G1V 0A6, Canada; 2 École de Nutrition, Université Laval, Québec, QC G1V 0A6, Canada

**Keywords:** food well-being, health, pleasure, questionnaire, validation, adults, Canada

## Abstract

Efforts to develop effective strategies that improve dietary intake are needed; however, this improvement in diet quality must not be at the expense of well-being. The Well-Being related to Food Questionnaire (Well-BFQ©) is a tool that has been developed in France to comprehensively measure food well-being. Even though the same language is spoken in France and in Québec, cultural and linguistic differences are present, which supports the importance of adapting and validating this tool before its use in the Québec population. This study aimed to adapt and validate the Well-BFQ© for the French-speaking general adult population of Québec, Canada. The Well-BFQ© underwent a full linguistic adaptation process, including an expert panel adaptation step, a pretest among 30 French-speaking adult (18–65 years) Quebecers, and a final proofreading. The questionnaire was thereafter administered to 203 French-speaking adult Quebecers (49.3% females, M_AGE_ = 34.9, SD = 13.5; 88.2% Caucasians; 54.2% with a university degree). The exploratory factor analysis showed a two-factor structure: (1) food well-being related to physical and psychological health (27 items) and (2) food well-being related to symbolic/pleasure of food (32 items). Internal consistency was adequate, with a Cronbach’s α of 0.92 and 0.93, respectively, for the subscales, and 0.94 for the total scale. The total food well-being score, as well as the two subscale scores, were associated with psychological and eating-related variables in expected directions. Overall, the adapted version of the Well-BFQ© was found to be a valid instrument to measure food well-being in the French-speaking general adult population of Québec, Canada.

## 1. Introduction

It is widely recognized that healthy eating can prevent the development of chronic diseases and that a substantial proportion of cardiometabolic deaths is associated with a suboptimal diet [1,2,3,4]. Multiple disease states and their detrimental effects on morbidity and mortality can be prevented or minimized with effective dietary and lifestyle interventions [5,6]. This body of knowledge has contributed to the emergence of a “food as medicine” paradigm, where healthy eating is identified as an adequate food and nutrient intake to prevent, manage, and treat illness [7,8,9,10,11,12]. In this paradigm, some foods may be considered to possess medicinal qualities that provide health benefits beyond their basic nutritional contributions. Considering the “food as medicine” paradigm, various tools based on food and nutrient intake have been developed to measure diet quality [13,14]. However, in addition to providing the necessary nutrients, foods also have emotional, social, symbolic, and hedonic values [15]. Eating is more than the amount of food we eat; it is also about how we eat it. In this regard, a paradigm shift from “food as medicine” to “food as well-being” has been claimed by an increasing number of researchers and health professionals [16,17,18,19,20]. According to the World Health Organization definition of health, healthy eating could be more comprehensively defined as eating behaviors that can enable the person to achieve “a state of complete physical, mental, and social well-being and not merely the absence of disease or infirmity” [21]. Accordingly, the “food as well-being” paradigm considers the psychological and social dimensions of food consumption, and not merely the biomedical orientation of food consumption. This paradigm shift is not only appearing in the scientific literature, but also in recent dietary guidelines in some countries, who now offer many tips above and beyond the choice of what foods to eat [22,23]. As an example, in addition to food choices, the newest version of the Canada’s food guide now includes recommendations about healthy eating habits, such as being mindful about one’s eating habits, cooking more often, enjoying food, and eating meals with others [24].

Accordingly, efforts to develop effective strategies that improve dietary intake are urgently needed to enhance populational health; however, this improvement in diet quality must not be at the expense of well-being. Namely, restricting food intake may lead to a repetitive pattern of self-deprivation, which can result in disordered eating such as bingeing, lower self-esteem, and weight changes, including weight gain and worsening body dissatisfaction [25]. In this context, the use of food well-being (FWB) as an outcome, in addition to diet quality, is of great interest. Block et al. [16] defined FWB as a positive psychological, physical, emotional, and social relationship with food at both individual and societal levels. Even if nearly 100 self-reported measures of well-being have been developed over the past 50 years [26], few tools have been developed to measure FWB [27,28,29,30,31,32]. The development of comprehensive measurement tools and their validation could be useful in research to supplement information provided by diet quality indices, allowing for a broader measurement of the concept of healthy eating. One questionnaire that has been developed in France to measure FWB comprehensively is the Well-Being related to Food Questionnaire (Well-BFQ©) [32]. The 134-item questionnaire is divided into six thematic modules, i.e., “Grocery shopping”, “Cooking”, “Dining places”, “Commensality”, “Eating and drinking”, and “Eating habits and health”. To develop the structure of the questionnaire, 24 focus groups (198 subjects) were conducted with French subjects to collect qualitative data about their definition and experience of well-being in general and more specifically in the context of food and diet. Pleasure and health were the two major domains emerging from these discussions that subjects linked to FWB. After the development of the questionnaire, a preliminary validation was conducted on 444 French subjects. Using principal component analyses, the structure of the questionnaire was determined, with confirmation of the sub-sections “immediate benefits” (pleasure, security, relaxation), “direct short-term benefits” (digestion and satiety, energy and psychology), “deferred long-term benefits” (eating habits and health), and “food behaviors”. In total, thirty-three subscales and 15 single items were identified. Confirmatory factor analyses confirmed the structure, with overall moderate to excellent convergent and divergent validity and internal consistency reliability among the French population [32].

Even if the French population (France) and the French-speaking population in Québec (Canada) have a common ancestral and cultural background, food habits in Québec have been influenced by the North American culture [33]. Nowadays, the eating habits and food-related attitudes of Quebecers are somewhat different from those of the French [33,34,35]. In addition, there are numerous linguistic specificities related to food that differentiate these two nations. In this regard, guidelines recommend that questionnaires be adapted culturally to maintain their content validity at a conceptual level, even if the language is the same [36]. The adaptation and validation of an instrument to consider the cultural and linguistic specificities of a target population ensures that it will be more culturally relevant and easily understood by the people to whom the tool is administered. In this study, we thus aimed to adapt and validate the Well-BFQ©, which was initially developed for the French population (France) for the French-speaking general adult population of Québec, Canada. The structure of the scale was assessed using exploratory factor analysis. We expected to find a similar structure to the one observed for the original version of the questionnaire. The internal consistency and the construct validity of the Well-BFQ© were also assessed. 

Results indicate that the adapted and validated version of the Well-BFQ© is a suitable instrument which can be used to measure FWB in the French-speaking general adult population of Québec, Canada. More precisely, a two-factor structure was found, mainly FWB related to physical and psychological health and FWB related to symbolic/pleasure of food.

## 2. Materials and Methods

The Well-BFQ© underwent a full linguistic adaptation process, including an expert panel adaptation step, a pretest among French-speaking adult subjects from the province of Québec (Canada) and a final proofreading. These three linguistic validation steps were supervised by the authors of the original version of the questionnaire. These steps were followed by a validation study.

### 2.1. Expert Panel Adaptation

Three registered dietitians (A.B., L.C., C.T.-G.), who are native speakers of the target language (Québec French) and proficient in the source language (France French), first identified all items that were not suitable to the cultural or linguistic context of Québec (Canada) and suggested alternative formulations. The suggestions were thereafter reviewed by a nutrition researcher (S.L.) with extensive expertise in the development and translation of questionnaires, who decided, after a discussion with the three registered dietitians, if the suggested changes were appropriate.

### 2.2. Pretest

After the expert panel adaptation, 30 French-speaking participants (15 men and 15 women), aged between 18 and 65 years old, from the Québec City metropolitan area, were recruited to assess face validity of the adapted questionnaire (M_AGE_ = 47.2, SD = 11.9; 86.7% Caucasians; 63.3% with a university degree). They were recruited from an internal list of people willing to participate in clinical nutrition studies. Each participant provided informed written consent before they participated in the study. The study was conducted in accordance with the Declaration of Helsinki, and the pretest study was approved by the Research Ethics Committee of Université Laval (#2017-045; 2 May 2017).

Subjects were asked to complete the questionnaire on a secured online platform (FANI, http://inaf.fsaa.ulaval.ca/fani/) and to comment, using a comment box, each instruction and item of the questionnaire (e.g., ease of completion, comprehension problems, alternative wording for problematic items). The expert panel reviewed the comments and made adjustments to improve the acceptability and comprehension of the problematic instructions/items.

### 2.3. Proofreading

To resolve any typing, spelling, or grammatical mistakes, the expert panel reviewed the final version of the questionnaire. They then sent the final proofread version of the questionnaire to the authors of the original questionnaire for approval of the modifications performed.

### 2.4. Validation Study

The validation of the Well-BFQ© was achieved within the context of a prior study, which took place between September 2017 and February 2018, and that has been described previously [37]. A total of 213 French-speaking adults (110 men and 103 women) from the Québec City metropolitan area were recruited. Participants had to be aged between 18 and 65 years old and had to perceive that their food habits needed to be improved. Participants were excluded if they met the following exclusion criteria: (1) having dietary behaviors that could significantly affect food choices (eating disorders, vegetarianism), (2) having food allergies or intolerance, (3) working or studying as a nutritionist/registered dietitian, (4) being pregnant or breastfeeding women, or (5) having participated in an intervention study about nutrition in the previous six months. Each participant provided written informed consent before they participated in the study. The study was conducted in accordance with the Declaration of Helsinki, and the protocol was approved by the Research Ethics Committee of Université Laval (#2017-146; 3 July 2017).

#### 2.4.1. Questionnaires

All questionnaires were completed on the same secured online platform as the pretest (FANI, http://inaf.fsaa.ulaval.ca/fani/). Respondents were first asked to complete the adapted 134-item version of the Well-BFQ©. At the same time, participants also completed other questionnaires presented below.

A 29-item questionnaire was used to collect socio-demographic data (e.g., sex, age, ethnicity, marital status, education level, and annual household income).

A 26-item questionnaire was used to document participants’ medical antecedents. In this questionnaire, nine specific medical antecedents were directly assessed (i.e., diabetes, cardiovascular diseases, hypertension, dyslipidemia, thyroid gland disorder, gastrointestinal disorders, liver diseases, kidney diseases, and cancer). The last section of the questionnaire asked participants to report other health problems not previously mentioned, if any.

Food preoccupation was measured using the following true/false question: “Do you consider yourself to be concerned about food?”.

The validated orientation to happiness scale was used to measure individuals’ orientations to happiness through the pursuit of pleasure, engagement, and meaning [38,39]. The scale contains a total of 18 items rated on a 5-point scale, from 1 = ‘very much unlike me’ through 5 = ‘very much like me’. A total score was calculated, with a higher score indicating a higher orientation to happiness.

The Health and Taste Attitudes Questionnaire has been developed and validated by Roininen et al. [40] to assess consumers’ orientations toward the health and hedonic characteristics of foods. This questionnaire included 38 items rated on a 7-point Likert scale, from 1 = ‘strongly disagree’ through 7 = ‘strongly agree’. Reversed scoring was applied to negatively worded items. The health-related factor labelled “General health interest” (8 items) was used to measure the health orientation of participants, while the taste-related factor named “Pleasure” (6 items) measured the pleasure orientation. A higher score represents a higher orientation toward health or pleasure.

The validated 24-item Regulation of Eating Behavior Scale was used to assess the type of motivation used for the regulation of eating behaviors according to the self-determination theory [41,42]. Items included in this questionnaire are rated on a 7-point Likert scale and assess, among others, intrinsic motivation and amotivation. A higher score indicates a higher level for each type of motivation.

Three web-based 24-h food recalls were completed using an online application developed by our research team, the R24W, which was specifically developed and validated for the French-speaking population of Québec, Canada [43,44,45]. Data generated by the dietary recalls were used to calculate the Canadian adapted Healthy Eating Index (C-HEI). The C-HEI is a measure of diet quality based on recommendations of the 2007 Canada’s food guide, which was the food guide in effect at the time of the study [40]. It is composed of eight adequacy components, including total fruits and vegetables, whole fruits, dark-green and orange vegetables, grain products, whole-grain products, milk and alternatives, meat and alternatives, and unsaturated fat, and three moderation components, including saturated fat, sodium, and “other foods” that are not part of the foods recommended by Canada’s Food Guide. The total score can range from 0 to 100. A total score of less than 50 was considered as a poor diet, a score of 50 to 80 was considered a diet requiring improvement, and a score of more than 80 was considered a good diet.

The Three-Factor Eating Questionnaire (TFEQ) is a 51-item validated questionnaire used for measuring three cognitive and behavioral components associated with eating, namely cognitive dietary restraint, disinhibition, and susceptibility to hunger [46]. The first concept refers to cognitive dietary restraint (Restraint; on a scale of 0 to 21 points), i.e., conscious control of food intake with concerns about body shape and weight. The second refers to disinhibition (Disinhibition; on a scale of 0 to 16 points), i.e., overconsumption of food in response to a variety of stimuli associated with a loss of control over food intake. The third concept is susceptibility to hunger (Hunger; on a scale of 0 to 14 points), i.e., food intake or eating in response to feelings and subjective perceptions of hunger. This questionnaire is divided into two parts, the first part consisting of 36 true/false questions and the remaining 15 items rated on 4- or 5-point Likert scales. A higher score indicates a higher level for each eating behavior evaluated.

According to standardized procedures, the research team measured height to the nearest millimeter with a stadiometer and body weight to the nearest 0.1 kg on a calibrated balance [47]. Body mass index (BMI; kg/m^2^) was then calculated.

#### 2.4.2. Statistical Analyses

The structure of the scale was assessed using exploratory factor analysis (EFA), with pairwise treatment for missing cases. As the participant to item ratio was below 5:1, the ULS method was used [48]. EFA was chosen instead of principal component analysis (PCA) or confirmatory factor analysis (CFA) because we wanted to explore the possible underlying factor structure in our target population without imposing any preconceived structure on the outcome [49]. To assess the number of factors to retain for the structure, the eigenvalue-greater-than-one rule, the analysis of the variance explained, and the scree plot were used. More specifically, only factors with an eigenvalue of 1 or greater and with a variance of more than 5% were retained [50]. According to the scree plot, the “elbow” of the graph where the eigenvalues seem to level off was found and factors to the left of this point were retained as significant [50]. Items with a contribution ≤0.4 on all factors, as well as items with a contribution > 0.4 on at least two factors, were eliminated. Once this first step was completed, EFA were performed again based on the remaining items, and adjustments were made in successive iterations to achieve the final structure of the questionnaire. Internal consistency was assessed with Cronbach’s α coefficients.

Once the final structure of the questionnaire was defined, a total score, as well as a score for each factor, were calculated by adding up the scores of each scale’s items. Each item was rated on a 5-point scale from 0 = ‘never’ to 4 = ‘always’ and negative items were reverse-coded. All scores were linearly transformed to be presented on a scale from 0 to 100. Higher scores indicated higher FWB. The score was not calculated if participants completed less than 66% of items included in the scale/subscale. Floor or ceiling effects were considered to be present if more than 15% of participants achieved the lowest or highest possible scale score, respectively [51]. If floor or ceiling effects were present, it was likely that extreme items were missing in the lower or upper end of the scale, indicating limited content validity for our population [51].

To assess construct validity, Pearson’s correlation analyses were conducted to investigate the association between Well-BFQ© scores and various psychological and eating-related variables, namely happiness orientation, pleasure and health orientations toward food, motivations for regulating eating behaviors (intrinsic motivation and amotivation), diet quality (C-HEI), as well as eating behaviors (cognitive dietary restraint, disinhibition, susceptibility to hunger), which are expected to be associated with the concept of FWB. Happiness has been identified as a central component of well-being in previous research [52]. In addition, since health and pleasure are the two main factors of FWB identified in the conceptual model of Guillemin et al. [32] used to develop the Well-BFQ©, health and pleasure orientations toward food should also be positively associated with the Well-BFQ© scores. With regard to motivation types, according to the self-determination theory, FWB should be positively associated with intrinsic motivation (which refers to engaging in an activity for its own sake and experience of pleasure and satisfaction derived from participation) and negatively associated with amotivation (which pertains to the lack of intentionality and therefore refers to the relative absence of motivation) to regulate eating behaviors [53]. Finally, well-being has also been previously associated with better diet quality (e.g., higher C-HEI, higher intake of fruits and vegetables), as well as with healthy eating behaviors (e.g., lower disinhibition and susceptibility to hunger) [54,55,56]. The classification of Cohen, i.e., a correlation coefficient of 0.1 being classified as small, of 0.3 being classified as moderate and of 0.5 being classified as strong, was used to interpret effect size [57].

A known-group approach was also used to measure construct validity. Differences in Well-BFQ© scores between subgroups were assessed using the Student’s *t*-test procedure (for two subgroups; variables: sex (men/women), medical antecedents (yes/no) and preoccupation toward food (yes/no)) and the generalized linear model (GLM) procedure (for more than two subgroups; variables: age and BMI). According to the literature related to well-being, FWB should be negatively affected by food preoccupation [58], BMI [59,60,61], and the presence of medical antecedents [62], and positively associated with age [63] and not affected by sex [64,65].

All statistical analyses were performed using the SAS software, version 9.4 (SAS Institute Inc., Cary, NC, USA). A *p* < 0.05 was considered significant.

## 3. Results

### 3.1. Adaptation Steps

#### 3.1.1. Expert Panel

The expert panel identified one title section, two section instructions, and 24 items that were not suitable to the cultural or linguistic context of our population. Problems identified were due to (1) expressions rarely used in Québec, (2) expressions with different meanings in France and in Québec and (3) wording that may not be clear for the target population. The main changes to the questionnaire made by the expert panel are listed in Table 1.

#### 3.1.2. Pretest

Face validity of the scale was assessed by pretest participants who formulated comments about the acceptability and comprehension of the scale. Based on the participants’ comments, three items were modified. The problems were due to words that were confusing and/or not clear for our population. We also added some explanations to certain items that were unclear for the participants. The main changes made following the pretest are also summarized in Table 1.

#### 3.1.3. Proofreading

The expert panel proofread the final version of the questionnaire. No typing, spelling, or grammatical mistakes were found.

### 3.2. Validation Study

Of the 213 participants, ten (seven men and three women) were excluded from the analyses since they dropped out before the completion of the Well-BFQ©. Table 2 shows descriptive characteristics of participants. Participants included in this study were men and women (women: 49.3%; men: 50.7%), with a mean age of 34.9 (SD = 13.5) and a mean BMI of 26.2 kg/m^2^ (SD = 5.5), mostly Caucasians (88.2%) and singles (53.2%). The majority of participants had a household income of less than $60,000 (53.7%) and a university degree (54.2%). Mean C-HEI score was 54.1 (SD = 13.6), indicating a diet requiring improvement.

Mean time for completion of this 134-item questionnaire was 20 min (SD = 15). A total of 154 participants (76%) responded to all items of the questionnaires. Thirty-nine items showed one missing data (0.49%), 12 items showed two missing data (0.99%), three items showed three missing data (1.48%), and one item showed four missing data (1.97%).

#### 3.2.1. Exploratory Factor Analysis

The EFA was performed on the 134 questionnaire items. The result from the Kaiser–Meyer–Olkin test of sampling adequacy was more than 0.5 (measure of sample adequacy = 0.57), a value that is considered suitable for factor analysis [66,67,68]. The significance of Bartlett’s test of sphericity was also considered suitable for factor analysis (khi-2 = 18694.5833, *p* < 0.0001) [66,67]. These two results justified the use of an EFA given the common variance of the set of items.

Thirty-one factors were retained with eigenvalues greater than 1. However, the explained percentage variance showed a two-factor structure with a variance in the data of more than 5% for only two factors (O’Rourke and Hatcher, 2013), accounting, respectively, for 16.5% and 9.7% of the variance. In addition, according to the scree plot, the “elbow” of the graph identified only two significant factors. It was therefore decided to use a two-factor solution for the structure of the questionnaire. We made a factor rotation to help the interpretation of the factor structure. In order to decide between an orthogonal and an oblique rotation, an oblique (promax) rotation was first requested to obtain a correlation matrix. Because the correlation between the two factors (r = 0.27) was below 0.32 [67], we decided to use an orthogonal (varimax) rotation, which was more suitable considering the structure of the questionnaire.

Using a cut-off value of 0.4 for factor loading, we removed 75 items from the questionnaire as they loaded too poorly on both factors. No retained item loaded simultaneously on the two factors. The final scale consisted of two subscales: one related to the “Physical and psychological health” (27 items; factor 1) and the other related to the “Symbolic/pleasure of food” (32 items; factor 2). The first factor refers to the impact that psychological and physical health can have on FWB, while the second factor refers to the symbolic value of food and pleasure that we can derive from it. Factor loadings and items are presented in Table 3.

For these two factors, none of the participants had the highest or the lowest score, suggesting no floor and ceiling effects (score range: factor 1: 7.4 to 99.1; factor 2: 10.9 to 93.8). The same result was observed for the total score (score range: 10.6 to 82.6). These results indicate that scales used in this questionnaire are sensitive in capturing the variation in FWB in our target population. No participant completed less than 66% of the items included in each scale/subscale, allowing for scale/subscale scoring for each participant.

#### 3.2.2. Internal Consistency

After removing items that loaded poorly on both factors, internal consistency was adequate, with Cronbach’s α coefficient of 0.92 for factor 1 (i.e., FWB-related physical and psychological health), 0.93 for factor 2 (i.e., FWB-related to the symbolic/pleasure of food), and 0.94 for all the retained items (see Table 3).

#### 3.2.3. Construct Validity

Table 4 shows Pearson correlations between Well-BFQ© scores and psychological and eating-related variables. The total FWB score, as well as the two subscales, were positively associated with happiness orientation (small to moderate correlations; r = 0.29 to 0.39, *p* < 0.0001), health orientation toward food (moderate correlations; r = 0.32 to 0.40, *p* < 0.0001), intrinsic motivation for regulating eating behaviors (moderate to strong correlations; r = 0.44 to 0.59, *p* < 0.0001), and C-HEI (small to moderate correlations; r = 0.19 to 0.30, *p* ≤ 0.007), and were inversely associated with amotivation for regulating eating behaviors (small correlations; r = −0.18 to −0.23, *p* ≤ 0.01). The FWB total score and the FWB related to symbolic/pleasure subscale score were also positively associated with pleasure orientation toward food (small to moderate correlations; r = 0.29, *p* < 0.0001 and r = 0.38, *p* < 0.0001, respectively). For eating behaviors, total FWB score and the FWB related to physical and psychological health subscale score were both inversely associated with susceptibility to hunger (small to moderate correlations; r = −0.16, *p* = 0.03 and r = −0.30, *p* < 0.0001, respectively), whereas only the FWB related to physical and psychological health subscale score was negatively associated with disinhibition (small correlation; r = −0.24, *p* = 0.0005).

FWB total and subscale scores were similar between age groups (Table 5). However, FWB related to physical and psychological health subscale score was higher in men than in women (*p* = 0.02). The total FWB score and the FWB related to physical and psychological health subscale score were both significantly lower in participants with obesity than in normal weight and overweight participants (*p* = 0.003 and *p* < 0.0001, respectively). Furthermore, those with medical antecedents reported lower FWB on the physical and psychological health subscale than those without personal medical antecedents (*p* = 0.01). In addition, participants who considered themselves as being preoccupied with food tended to report a lower total FWB score and FWB related to physical and psychological health subscale score than those not preoccupied about food (*p* = 0.08 and *p* = 0.06, respectively).

## 4. Discussion

The Well-BFQ© is a questionnaire that has been developed in France to comprehensively measure FWB [32]. Despite the fact that the same language (i.e., French) is spoken in France and in Québec, cultural and linguistic differences exist between these two populations [34,69]. This supports the importance of adapting and validating the Well-BFQ© before its use in Québec, Canada. In this study, we first modified the Well-BFQ© to obtain a version that is culturally relevant and easily understood by our adult population, which was followed by a validation study. Results indicate that the adapted version of the Well-BFQ© has good psychometric properties, and thus that this is a suitable instrument which can be used to measure FWB in the French-speaking adult population of Québec, Canada.

We obtained a different questionnaire structure from the one observed in the original validation study. In fact, Guillemin et al. [32] reported a 7-factor structure in the French population, namely “immediate pleasure benefits”, “immediate security benefits”, “immediate relaxation benefits”, “direct digestion and satiety benefits”, “direct energy and psychology benefits”, “well-being food behaviors”, and “deferred health benefits”. In the present validation study, however, we observed a two-factor structure related to FWB: “Physical and psychological health” and “Symbolic/pleasure of food”. It is worth mentioning that different analyses were conducted in these two validation studies (principal component analysis in the previous study and exploratory factor analysis in the present study), which may partly explain the differences observed in the structure of the questionnaire. However, this two-factor structure is in concordance with the two main factors of FWB that have been previously identified in the conceptual model of Guillemin et al. [32], and which served as the basis for the development of the Well-BFQ©. In fact, their analysis using focus groups with 198 French subjects indicated that FWB articulates around two central domains that are health and pleasure. These results are also in line with those of Ares et al. [52], suggesting that the influence of food on well-being is strongly associated with physical and psychological health as well as with pleasure. In our study, the “Physical and psychological health” factor contains several items related to physical health (e.g., back, joints, bones, arteries, immune system), which provides a fairly comprehensive measure of this concept. Although they are fewer in number, it also contains items documenting the impact of eating habits on mental health, such as how eating habits impact individuals’ morale and feelings. Interestingly, this factor allows for documenting immediate and direct health benefits of eating habits (e.g., energy throughout the day, digestion, breathing), but also for deferred health benefits (e.g., life expectancy, bone strength, avoidance of cholesterol- and diabetes-related problems). For the “Symbolic/pleasure of food” factor, some items directly document the pleasure that we can derive from food (e.g., going shopping, preparing meals, discovering new foods), while some others are related to values that people may have about foods (e.g., eating local food, fresh food, seasonal food, organic food, and food of known origin/provenance). Overall, the Well-FBQ© presented good psychometric properties in our population. In fact, internal consistency for each of the two factors is considered excellent (≥0.92), indicating that items in each subscale are strongly correlated and support the structure of the questionnaire. Internal consistency for the total score was also excellent, which means that the questionnaire measures a single overall concept and can be used to measure a total score of FWB.

To evaluate the construct validity of the questionnaire, we investigated the associations between FWB scores and some psychological and eating-related outcomes that are expected to be associated with the concept of well-being. First, we found moderate to strong associations between FWB scores and intrinsic motivation to regulate eating behaviors, with participants with greater FWB being characterized by a higher level of intrinsic motivation. These results are in line with the self-determination theory that suggested that well-being is increased when individuals present intrinsic motivation for regulating their eating behaviors [53]. Intrinsic motivation is defined as the doing of an activity for its own sake and experience of pleasure and satisfaction derived from participation rather than from expectations about other consequences [53]. Thus, participants with this type of motivation seem more likely to make food choices for their own satisfaction and pleasure, which may in turn increase the FWB experienced. Higher FWB scores were also associated with a lower amotivation to regulate eating behaviors, i.e., a lack of intentionality due to the relative absence of motivation. This lack of motivation can result in individuals not changing eating behaviors, even if they are not comfortable with them. In addition, we found small to moderate associations between FWB and the health and pleasure orientations toward food. These results are in line with the structure of the scale, with the two main factors of FWB being health and pleasure. As expected, the physical/psychological health subscale was positively associated with the health orientation toward food. However, the symbolic/pleasure subscale was positively associated with both health and pleasure orientations toward food. These results may be explained by the fact that the symbolic/pleasure subscale has many items related to the importance of pleasure, but also items related to the symbolic of food, including some that may be associated with better health (e.g., importance to eat fresh, local, organic and seasonal foods). A higher FWB was also slightly to moderately associated with a greater orientation to happiness, which is known as a central component of well-being [70].

Results also showed that higher FWB scores were slightly to moderately associated with a better diet quality, as measured by the C-HEI. Research suggests that the levels of well-being can influence our responses to food [71]. However, foods may also affect the consumer’s perceived well-being [52,54,56]. In a study by Ares et al. [54], participants reported that foods that have a favorable impact on well-being were those recognized as being healthy, such as fruits and vegetables, while fatty, fried, and sugary food were perceived as reducing well-being. In addition, Holder [56] reviewed the contribution of food consumption to well-being. This paper suggested that healthy eating, particularly the intake of fruits and vegetables, is associated with higher levels of well-being, and that an increased fruit and vegetable intake results in increased well-being in a dose–response fashion. Taken together, these results are in line with ours, suggesting a link between well-being and diet quality. In addition, in our study, small to moderate associations have been observed between FWB and eating behaviors, suggesting that FWB is linked to more healthy eating behaviors (lower levels of susceptibility to hunger and disinhibition). This is consistent with findings from Provencher et al. [72], which showed a significant negative relationship between psychological well-being, and disinhibition, susceptibility to hunger, and all their subscales in a population of postmenopausal women using the same questionnaire as we did (Three-Factor Eating Questionnaire). Overall, these results are supportive of a good construct validity of the scale, since associations with psychological and eating variables occurred in expected directions.

Regarding subgroups analyses, the total score and subscale scores were similar between age groups. Comparison with other studies is difficult, considering that research about the concept of FWB is still at a nascent stage. A U-shape association is generally observed between well-being and age groups [63]; however, the relatively narrow age range limit used in the present study may have impeded the observation of this association. Results were also similar between men and women, except for FWB related to physical and psychological health, with men reporting higher score than women. These results are in line with the literature, suggesting no clear sex differences related to the general concept of well-being [64,65]. We also found that those with medical antecedents reported lower FWB related to physical and psychological health subscale score than those without personal medical antecedents. These results are in accordance with those of Stewart et al. [62], which showed adverse effects of chronic diseases on most aspects of functioning and well-being. Total FWB and FWB related to physical and psychological health scores were significantly lower for participants with obesity compared to normal weight and overweight participants. In addition, participants who considered themselves as being preoccupied with food also tended to report lower scores for these two scales. A culture based on worry and preoccupation with weight and food can have detrimental effects on well-being [73]. Concern about weight may lead to preoccupation with weight gain and appearance, and behaviors such as dieting [58,74], which may thereafter impede FWB. As previously suggested, obsessive thoughts about food and eating may also lead to a lower well-being [58].

The major strengths of the study include the rigorous adaptation process of the questionnaire for the target population, which was based on a three-step method proposed by the developer of the Well-BFQ©. Another strength was that we recruited a well-distributed sample in terms of sex, age, and BMI. However, this study also has some limitations. The sample size may be viewed as slightly small. Generally, EFA procedures require fairly large sample sizes. However, although different authors give different guidelines, it is well accepted that a minimum of 100 participants is required [75]. Therefore, considering these guidelines, our sample size of more than 200 participants is adequate. Nevertheless, a confirmatory factor analysis should be performed to confirm the structure of the questionnaire in our population in the next study. Additionally, a test–retest should be done in order to evaluate whether this questionnaire could be reliably replicated more than once in the same situation and population.

## 5. Conclusions

The adapted and validated version of the Well-BFQ© was found to be a suitable instrument, which can be used to measure FWB in the French-speaking general adult population of Québec, Canada. Results showed a two-factor structure, namely (1) FWB related to physical and psychological health and (2) FWB related to symbolic/pleasure of food. Internal consistency was adequate, and the total food well-being score, as well as the two subscale scores, were associated with psychological and eating-related variables in expected directions, highlighting the good construct validity of the scale. The fact that recent Canadian dietary guidelines now offer a more comprehensive view of healthy eating, including recommendations not only about healthy food choices but also about a healthy relation with food, underlines the relevance of validating tools such as the Well-BFQ© in the Canadian population. Therefore, the validation of the Well-BFQ© sets an exciting path for researchers, allowing for the combination of diet quality and this FWB tool to comprehensively measure the concept of healthy eating in different segments of our population. In addition, this will also allow for the comparison of FWB in response to different nutritional interventions, thereby leading to the identification of those interventions that favor both better diet quality and higher FWB.

## Figures and Tables

**Table 1:** Main changes made to the questionnaire during the adaptation process.

Types of Modifications	Modifications Made to the Questionnaire	Reasons for the Changes
**Step 1: Expert panel**		
Reformulation of section title	Replace “faire ses courses” with “faire ses achats alimentaires” in Section 1.	Expression rarely used in Québec
Reformulation of section instructions	Replace “fait vos courses” with “fait vos achats alimentaires” in Section 1. Replace “compléments alimentaires” with “suppléments alimentaires” in Section 6 “Habitudes alimentaires et santé”.	Expression rarely used in Québec Expression rarely used in Québec
Reformulations and restructuration of items	Replace “fait mes courses” with “fait mes achats alimentaires” in items 1, 2 and 3. Replace “faire ses courses” with “faire ses achats alimentaires” in items 5 and 8. Replace “Quand je fais mes courses” with “quand je fais mes achats alimentaires” in items 9, 10 and 11. Replace “Ustensiles et matériel de cuisson” with “accessoires et équipements de cuisson” in item 13. Replace “plats tout prêts” with “repas prêts-à-manger” in items 15 and 62. Replace “pour un repas de fête sans invités” with “pour un repas de fête, en famille” in items 24, 25 and 26. Replace “ce qui me fait envie” with “ce dont j’ai envie” in item 57. Replace “compléments alimentaires” with “suppléments alimentaires” in item 70. Replace “coups de pompe” with “baisses d’énergie” in item 96. To add an explanation at the end of the item 102, i.e., “j’ai des remontées acides (acidité dans la bouche)”. Replace “je me sens barbouillé(e)” with “je me sens nauséeux(se)” in item 103. To add an explanation at the end of the item 105, i.e., “j’ai l’impression de remanger certains aliments toute la journée (avoir des renvois après avoir mangé) ”. To add an explanation at the end of the item 106, i.e., “je me sens écœuré(e) (je n’ai absolument plus envie de manger) ”. Replace “je me sens lourd” with “j’ai l’impression d’avoir trop mangé(e)” in item 107. Replace “habits” with “vêtements” in item 111. Replace “avoir bonne mine” with “avoir l’air bien” in item 116.	Expression rarely used in Québec Expression rarely used in Québec Expression rarely used in Québec Different meaning in France and in Québec Different meaning in France and in Québec Wording not clear for our population Expression rarely used in Québec Expression rarely used in Québec Expression rarely used in Québec Wording not clear for our population Expression not commonly used in Québec Wording not clear for our population Wording not clear for our population Expression not commonly used in Québec Expression not commonly used in Québec Expression not commonly used in Québec
**Step 2: Pretest**		
Reformulations and restructuration of items	Add “ambiance chaleureuse et agréable” at the end of the item 40 “J’accorde de l’importance à ce que les repas soient conviviaux”. Replace “lourd” with “plein” in item 53. Replace “analyses de sang” with “résultats sanguins” in item 113.	Some participants asked for the definition of the word “conviviaux” Some participants highlighted that the word “lourd” can be confusing Item not clear for some participants

**Table 2:** Participants’ characteristics.

Variables	Mean ± SD or *n* (%)	Range
**Sex**		
Women	100 (49.3)	
Men	103 (50.7)	
**Age (years)**	34.9 ± 13.5	18–65
**BMI (kg/m^2^) ^a^**	26.2 ± 5.5	18.4–54.1
Normal weight	106 (52.2)	
Overweight	62 (30.5)	
Obesity	35 (17.2)	
**Race/ethnicity**		
Caucasian	179 (88.2)	
**Marital status**		
Single	108 (53.2)	
Common-law union	54 (26.6)	
Married	28 (13.8)	
Divorced	7 (3.4)	
Separated	6 (3.0)	
**Highest education level**		
Elementary school	1 (0.5)	
High school	21 (10.3)	
CEGEP ^b^	70 (34.5)	
University	110 (54.2)	
I prefer not to answer	1 (0.5)	
**Household income**		
$0–19,999	49 (24.1)	
$20,000–39,999	30 (14.8)	
$40,000–59,999	30 (14.8)	
$60,000–79,999	24 (11.8)	
$80,000–99,999	16 (7.9)	
>$100,000	33 (16.3)	
I prefer not to answer	21 (10.3)	
**C-HEI score**	54.1 ± 13.6	23.3–88.9

*n* = 203; BMI: Body mass index; C-HEI: Canadian adapted Healthy Eating Index. ^a^ Normal weight <25.0 kg/m^2^; overweight 25.0–29.9 kg/m^2^; obesity ≥30.0 kg/m^2^. ^b^ In the Québec education system, CEGEP is the first level of postsecondary studies and precedes university studies. It includes pre-university programs and technical programs.

**Table 3:** Exploratory factor analysis with orthogonal rotation and descriptive analysis.

	Factor		Descriptive Analyses				Cronbach α
	Factor 1: Physical and Psychological Health	Factor 2: Symbolic/ Pleasure	Min	Max	Mean	SD	
**Factor 1**							0.92
52. My diet is balanced.	0.64		0	4	2.22	0.78	
57. I eat whatever I want even if I know I shouldn’t due to health reasons.	−0.46		0	4	2.15	0.79	
92. After eating, I feel in shape to perform my activities.	0.43		0	4	2.34	0.76	
111. After eating, my clothes feel tight.	−0.42		0	4	1.52	1.06	
112. I think that my eating habits are … for my daily health.	0.74		0	4	2.15	0.92	
113. I think that my eating habits are … for my blood test results.	0.65		0	4	2.14	0.84	
114. I think that my eating habits are … for my energy throughout the day.	0.63		0	4	2.33	0.86	
115. I think that my eating habits are … to keep my weight stable.	0.60		0	4	1.98	1.02	
116. I think that my eating habits are … to have a healthy glow.	0.74		0	4	2.18	0.93	
117. I think that my eating habits are … for my digestion.	0.67		0	4	2.20	0.83	
118. I think that my eating habits are … for my intestinal transit.	0.62		0	4	2.19	0.80	
119. I think that my eating habits are … to eliminate.	0.59		0	4	2.21	0.76	
120. I think that my eating habits are … for my joints.	0.60		0	4	2.09	0.66	
121. I think that my eating habits are … for my back.	0.54		0	4	2.08	0.61	
122. I think that my eating habits are … for my morale.	0.41		0	4	2.65	0.83	
123. I think that my eating habits are …to feel good in my mind and body.	0.57		0	4	2.47	0.91	
124. I think that my eating habits are … for my breathing.	0.72		0	4	2.07	0.75	
125. I think that my eating habits are … to slow down my ageing.	0.77		0	4	1.95	0.78	
126. I think that my eating habits are … to increase my life expectancy.	0.75		0	4	1.93	0.91	
127. I think that my eating habits are … to keep my skin young looking.	0.69		0	4	2.00	0.65	
128. I think that my eating habits are … to strengthen my bones.	0.57		0	4	2.35	0.82	
129. I think that my eating habits are … to keep my arteries from clogging.	0.68		0	4	2.08	0.86	
130. I think that my eating habits are … to avoid having cholesterol problems.	0.66		0	4	2.12	0.89	
131. I think that my eating habits are … to avoid having diabetes problems.	0.67		0	4	2.12	0.92	
132. I think that my eating habits are … to help me strengthen my immune system.	0.70		0	4	2.33	0.78	
133. I think that my eating habits are … to help me be sick less often.	0.73		0	4	2.34	0.80	
134. I think that my eating habits are … to help me fight diseases, viruses, bacteria.	0.71		0	4	2.33	0.78	
**Factor 2**							0.93
1. I go shopping where there are local food products.		0.51	0	4	1.96	0.85	
2. I go shopping where I can find seasonal food products.		0.51	0	4	2.13	0.92	
4. I grant importance to the origin of food products.		0.53	0	4	2.09	1.07	
5. Going shopping for food products brings me pleasure.		0.45	0	4	2.40	0.98	
7. Buying local food products brings me pleasure.		0.67	0	4	2.33	1.13	
8. Going shopping food products in places that break from my routine brings me pleasure.		0.56	0	4	2.24	1.16	
9. When I go shopping, it reassures me to buy local food products.		0.59	0	4	2.24	1.16	
10. When I go shopping, it reassures me to buy organic food products.		0.49	0	4	1.80	1.28	
11. When I go shopping, it reassures me to buy food products whose origin is known.		0.57	0	4	2.55	1.13	
12. When I prepare meals, I try to preserve the vitamins in foods.		0.41	0	4	2.05	1.10	
15. I prefer preparing meals myself rather than buying ready-to-eat meals.		0.43	0	4	2.89	1.00	
16. Preparing meals from day to day brings me pleasure.		0.62	0	4	2.13	0.96	
17. Preparing meals (for guests, for special occasions) brings me pleasure.		0.57	0	4	2.81	1.08	
18. When I cook, changing my habits brings me pleasure.		0.61	0	4	2.48	1.03	
19. When I cook, trying new recipes and new products brings me pleasure.		0.59	0	4	2.65	1.04	
20. When I prepare meals (for ordinary meals), it relaxes me.		0.57	0	4	2.02	1.00	
21. When I prepare meals (for ordinary meals), I feel good.		0.52	0	4	2.39	0.93	
22. When I prepare meals (for ordinary meals), it reassures me to have products I have cooked myself.		0.53	0	4	2.68	1.01	
23. When I prepare meals (for ordinary meals), I am satisfied.		0.48	0	4	2.91	0.71	
24. When I prepare meals (for holiday meals, with my family), it relaxes me.		0.52	0	4	2.02	1.14	
25. When I prepare meals (for holiday meals, with my family), I feel good.		0.57	0	4	2.56	1.04	
26. When I prepare meals (for holiday meals, with my family), I am satisfied.		0.47	0	4	2.74	0.93	
37. Eating my meals in places where I can discover new flavors or new dishes brings me pleasure.		0.44	0	4	3.09	0.81	
40. I grant importance to having convivial meals (warm, pleasant atmosphere).		0.41	0	4	3.00	0.90	
65. I make efforts to eat fresh products.		0.46	0	4	2.60	0.85	
66. I make efforts to eat organic products.		0.51	0	4	1.14	0.96	
67. I make efforts to eat seasonal products.		0.62	0	4	1.87	1.03	
68. I make efforts to eat local products.		0.62	0	4	1.77	0.95	
76. I grant importance to discovering new dishes, new recipes.		0.61	0	4	2.49	0.92	
79. Discovering new foods brings me pleasure.		0.53	0	4	2.90	0.79	
85. When I eat, it reassures me to know there are fresh products.		0.57	0	4	2.53	1.04	
86. When I eat, it reassures me to know there are organic products.		0.48	0	4	1.63	1.20	
**Total**							0.94

Items have been translated from French to English for the purpose of publication. However, only French items have been validated.

**Table 4:** Pearson’s correlations between food well-being scores and psychological and eating-related variables, and their means and standard deviations.

	Mean	SD	Physical and Psychological Health	Symbolic/ Pleasure	Total Food Well-Being
**Happiness**	3.48	0.43	0.29 ***	0.33 ***	0.39 ***
**Attitude toward food**					
Pleasure orientation	5.02	0.97	0.06	0.38 ***	0.29 ***
Health orientation	4.10	1.00	0.35 ***	0.32 ***	0.40 ***
**Motivation to regulate eating behaviors**					
Intrinsic motivation	17.7	5.5	0.44 ***	0.52 ***	0.59 ***
Amotivation	7.3	3.7	−0.18 *	−0.20 **	−0.23 **
**C-HEI**	54.1	13.6	0.30 ***	0.19 **	0.30 ***
**Eating behaviors**					
Restraint	6.69	3.89	0.11	0.02	0.07
Disinhibition	6.26	3.00	−0.24 ***	0.04	−0.11
Hunger	5.33	3.38	−0.30 ***	0.00	−0.16 *

*n* = 203; C-HEI: Canadian adapted Healthy Eating Index. Score range: Happiness = 1 to 5; Pleasure orientation = 1 to 7; Health orientation = 1 to 7; Intrinsic motivation = 4 to 28; Amotivation = 4 to 28; C-HEI = 0 to 100; Restraint = 0 to 21; Disinhibition = 0 to 16; Hunger = 0 to 14. * *p* < 0.05; ** *p* < 0.01; *** *p* < 0.001.

**Table 5:** Comparisons of food well-being scores according to sex, age, body mass index, medical antecedents, and preoccupation toward food.

Variable	*n*	Physical and Psychological Health	Symbolic/ Pleasure	Total Food Well-Being
**Sex**				
Women	100	52.5 ± 13.0	60.1 ± 14.4	56.6 ± 10.4
Men	103	57.0 ± 13.7	57.2 ± 14.1	57.2 ± 12.1
*p*-value		0.02	0.15	0.71
**Age**				
18–34 years	120	55.0 ± 13.5	58.0 ± 14.7	56.7 ± 11.5
35–49 years	42	53.4 ± 15.2	58.1 ± 14.0	55.9 ± 12.1
50–65 years	41	55.6 ± 11.7	61.0 ± 13.2	58.5 ± 9.6
*p*-value		0.72	0.49	0.55
**BMI**				
Normal	106	57.2 ± 12.6	59.8 ± 12.5	58.7 ± 9.9
Overweight	62	55.8 ± 12.1	58.1 ± 14.8	57.1 ± 11.0
Obesity	35	45.8 ± 15.0	55.8 ± 17.9	51.3 ± 13.9
*p*-value		<0.0001	0.34	0.003
**Medical antecedents**				
Yes	73	51.7 ± 13.3	58.9 ± 15.1	55.6 ± 11.2
No	130	56.6 ± 13.4	58.4 ± 13.8	57.6 ± 11.3
*p*-value		0.01	0.82	0.22
**Preoccupation toward food**				
Yes	114	53.2 ± 14.2	57.8 ± 14.9	55.7 ± 11.4
No	89	56.9 ± 12.4	59.6 ± 13.4	58.5 ± 10.9
*p*-value		0.06	0.37	0.08

*n* = 203; BMI: Body mass index. Differences between subgroups were assessed using the Student’s *t*-test procedure (for two subgroups; variables: sex (men/women), medical antecedents (yes/no) and preoccupation toward food (yes/no)) and the generalized linear model (GLM) procedure (for more than two subgroups; variables: age and BMI). Score range for each FWB scale = 0 to 100.

## Data Availability

The data underlying the findings are not publicly available because the original approval by the Research Ethics Committee of Université Laval and the informed consent from the subjects participating in the studies did not include such a direct, free access. Data can be requested from the principal investigator at simone.lemieux@fsaa.ulaval.ca for researchers who meet the criteria for access to confidential data.
